# Supplementary material for: Acceptance and compliance with micronutrient powder (MNP) among children aged 6–23 months in northern Nigeria
Source: PLOS Glob Public Health. 2022 Oct 17;2(10):e0000961. doi: 10.1371/journal.pgph.0000961 (PMC10022258; doi:10.1371/journal.pgph.0000961)
Supplement: S1 Dataset — (ZIP) [file pgph.0000961.s008.zip › S8_Dataset/In-depth Interviews.zip!/In-depth Interviews/010715_A_SO_BA_2_CG.pdf]

INDEPTH INTERVIEW (IDI) WITH MALLAM AHMADU OF BOLKI COMMUNITY, SONG L.G.A ON 01/07/2015 IN ADAMAWA STATE.

Introduction:

Mallam ahmadu has 12 children with 3 wives as members of his household. Presently only one child is legible and currently on MNP feeding. She is a girl of 6 months of age. The following were comments from mallam ahmadu regarding the use of MNP on her child:

Food for young children could be challenging atimes because of the nature of crops we cultivate here. But we have varieties of pap which I believe they are good for children to live healthy. For infants, these paps in varieties have proving to be nutritious and the young child is responding well and living healthy.

We don't give tea to the child feeding on MNP. We fed her on exclusive breastfeeding before 6 months and she is now on breastfeeding along with water and light food like pap and milk. But if you want us to be giving her, we'll comply.

Experience with MNP during trial:

The young child is feeding well and much more compare to before the MNP feeding. She's stronger and much healthy. The challenge we experienced at the onset with the MNP is the issue of diarrhea. My wife had wanted to stop using the product on her but I insisted she continue with it. Though it has subsided drastically. No barriers in using the product were observed. We give the MNP with pap in the morning hours and we've never skipped any day. We have no

challenges with storage. I store it myself and its free from any intruders or rodents. In general, the MNP is well kept. No drug was given to the child to treat diarrhea. I gave mallam ahmadu the assurance not to worry that it's a common side effect observed in some children at the start of the MNP and it will stop as they continue to take the MNP. The caregiver has no problem of forgetfulness. He said that they've never skipped any day without administering the MNP on the child. On caregivers having problems with forgetfulness, the spouse or husband should assist to remind her and give all the support to ensure usage on daily basis.

Child feeding behavior and response to MNP

The child is fed by mixing the MNP with pap and during morning hours. There is improved feeding by the child which may be related to the MNP or the child is getting use to the recent introduction into meal because previously it was totally exclusive breastfeeding. The taste has no negative effect on her feeding behavior. The MNP also increased her breastfeeding behavior.

Sharing MNP

No cases of sharing since we started using the MNP on the young girl because she is fed by the mother and the right quantity is given to her to prevent reminant. The caregiver said we should determine the right quantity of MNP needed by the child since we know much about the product than him on the child. They don't see daily administration of the MNP as a problem. But since the MNP has positive impact on the child, increasing it will be preferable.

Perception of the MNP

I see the MNP as a drug since it helps to improve the health of the child and much value is attached to it unlike food. Example, my other children are having slight fever but she's doing fine.

Willing to pay for MNP

Since MNP is improving the health of my child, I won't have problem paying for it. The seller should determine the cost since he know how much he's spent in production. Since it's vitamin supplement, if its to be bought the way drugs are sold, each sachet will cost 300 naira. But if I must pay for it, I can only afford 500 naira for the whole packet containing 30 sachets.

Social marketing of the MNP

People like the sarki (traditional head of the community) and religious leaders like imam and pastor influence my decisionand the community at large as well as doctors or health personnel on health issues such as MNP usage. I accepted the use of MNP for my daughter because I know it wont be harmful instead helpful for every child. I've never rejected programs aimed to improve human health. The best place to disseminate information on MNP is through mosque and churches. With the help of the religious leaders, hospitals and gaining support from traditional leaders, the MNP program can be lunched in the village square the gathering involving the community member. The hospital staffs are in the best position to disseminate information on health related issues. Through word by mouth from people like me who is a member of the community and has used the product on my child and have seen the positive impact can help disseminate the information and people will comply or accept

the product. Campaigns on vaccination against poliomyelitis were successful because they used health workers who were well trained along with adhoc staffs well known by the community for their activities. With the help from community leaders, they go house to house to carry out their activity. So distribution should be done house by house with help from religious leaders and not politicians. Messages to be communicated with people include, its for the benefit of their children and testimony from persons like him from the community who have used the product on his young child will be enough or adequate. Advice should be given on the importance of using the right quantity of kunu to mix the MNP to avoid remains and wastage of the MNP. Also its ideal to tell the people on importance of taking a full sachet of the MNP.

Final thought

My initial worry is the case of diarrhea but since you've addressed it, I'm fine with it. Also, on continuity, what are the plans of the management? My answer was, we are still consulting for now on ways to ensure continuity.
